# Incidence of renal injury following chemotherapy in malignant tumor patients and analysis of associated factors

**DOI:** 10.3389/fmed.2026.1758033

**Published:** 2026-04-17

**Authors:** Siyang Chen, Yutian Zhang, Juping Yang, Xijie Chen, Zhaohui Wang, Qunxiang Cao

**Affiliations:** 1 Department of Pharmacy, Affiliated Hospital of Xiangnan University, Chenzhou, Hunan, China; 2 Department of Pharmacy, The Affiliated Hospital of Jiangxi University of Chinese Medicine, Nanchang, Jiangxi, China; 3 Department of Digestion, The First People’ Hospital of Chenzhou, Chenzhou, Hunan, China

**Keywords:** chemotherapy, independent risk factors, malignant tumors, renal function indicators, renal injury

## Abstract

**Objective:**

To systematically investigate the clinical characteristics, peri-chemotherapy changes in renal function, and independent risk factors associated with chemotherapy-related renal injury (CRI) in patients with malignant tumors, providing scientific basis for developing individualized prevention and intervention strategies.

**Methods:**

A retrospective study design was adopted. A total of 152 patients with malignant tumors who underwent chemotherapy in the Department of Oncology of our hospital from January 2020 to December 2023 were included. Patients were categorized into a chemotherapy-related renal injury (CRI) group (*n* = 28) and a non-CRI group (*n* = 124) based on the Kidney Disease: Improving Global Outcomes (KDIGO) criteria. CRI was defined as a composite endpoint comprising either acute kidney injury (AKI) occurring within 1 week after the first chemotherapy cycle, or chronic kidney disease (CKD) persisting for more than 3 months post-chemotherapy. Renal function was assessed at standardized time points: within 1 week after cycle 1 and at the 3-month follow-up. The timing of post-chemotherapy renal function assessment was standardized for all patients, with the first evaluation conducted within 1 week following the completion of the first chemotherapy cycle (to capture AKI events) and a second evaluation performed at the 3-month follow-up visit (to assess CKD persistence). Patient demographics (age, gender, BMI, etc.), comorbidities (hypertension, diabetes, chronic kidney disease, etc.), tumor-related information (type, stage), chemotherapy regimens (use of nephrotoxic drugs, number of drugs), and renal function indicators before and after chemotherapy [serum creatinine (SCr), blood urea nitrogen (BUN), uric acid (UA), estimated glomerular filtration rate (eGFR)] were collected from the hospital electronic medical record system. Univariate analysis screened CRI-associated factors, followed by multivariate logistic regression to identify independent risk factors.

**Results:**

Among 152 patients undergoing chemotherapy for malignant tumors, the CRI incidence was 18.42% (28/152). The CRI group had a significantly higher proportion of patients aged ≥ 60 years, as well as higher rates of hypertension, diabetes, chronic kidney disease, use of nephrotoxic agents, and receipt of ≥ 3 chemotherapy drugs. in the CRI group than in the non-CRI group (all *P* < 0.05). Post-chemotherapy, the CRI group exhibited significantly elevated SCr, BUN, and UA levels and significantly reduced eGFR levels compared with the non-CRI group (all *P* < 0.001). Multivariate logistic regression analysis confirmed that age ≥ 60 years (OR = 3.277, 95% CI: 1.175–9.134, *P* = 0.023), concurrent diabetes (OR = 4.544, 95% CI: 1.612–12.809, *P* = 0.004), history of chronic kidney disease (OR = 6.348, 95% CI: 1.100–36.638, *P* = 0.039), use of nephrotoxic chemotherapy drugs (OR = 3.930, 95% CI: 1.143–13.511, *P* = 0.030), and increased number of chemotherapy drugs (OR = 2.068, 95% CI: 1.185–3.612, *P* = 0.011) were factors independently associated with CRI occurrence.

**Conclusion:**

This study identifies a relatively high incidence of CRI among patients with malignant tumors undergoing chemotherapy. The findings suggest that age ≥ 60 years, diabetes, chronic kidney disease, use of nephrotoxic agents, and an increased number of chemotherapy drugs are associated with higher risk of CRI. However, due to the retrospective design and limited number of CRI events, these findings should be interpreted with caution. Further prospective studies with larger sample sizes are needed to validate these associations. These associations highlight the need for enhanced renal function monitoring and cautious selection of chemotherapy regimens, particularly in high-risk populations.

## Introduction

The global incidence and mortality rates of malignant tumors continue to rise, establishing them as a leading threat to human health (1). As a core component of comprehensive cancer treatment, chemotherapy significantly prolongs survival in advanced-stage patients and improves clinical outcomes by inhibiting tumor cell proliferation and inducing apoptosis (2, 3). However, chemotherapeutic agents lack absolute tumor cell specificity. While killing tumor cells, they inevitably cause damage to normal tissues and organs. The kidneys, as the primary target organs for drug metabolism and excretion, are particularly susceptible to direct or indirect damage from chemotherapy drugs (4, 5).

Chemotherapy-induced renal injury (CRI) is a common severe adverse reaction during clinical chemotherapy. Its pathogenesis involves the combined effects of multiple intracellular stress responses, primarily including DNA damage, mitochondrial dysfunction, oxidative stress, and endoplasmic reticulum stress (6, 7). Certain chemotherapeutic agents, exemplified by cisplatin, demonstrate proven efficacy against various solid tumors such as lung and gastric cancers. However, their potent nephrotoxicity severely limits clinical application, potentially causing renal damage ranging from acute kidney injury to chronic kidney disease (8, 9). Studies indicate that CRI occurrence is closely associated with factors including tumor type, chemotherapy regimen, and patient baseline health status (10, 11). Mild CRI may present only as transient abnormalities in serum creatinine (SCr), blood urea nitrogen (BUN), and other indicators. Severe cases can progress to acute kidney injury (AKI), chronic kidney disease (CKD), or even end-stage renal disease. This not only necessitates interruption of chemotherapy regimens or dose adjustments, compromising tumor treatment efficacy, but also significantly increases patient infection risks, medical costs, and mortality (12, 13). Furthermore, patients with CRI exhibit substantially higher long-term risks of cardiovascular events and renal failure compared to those without renal injury, imposing a heavy burden on patient families and the healthcare system (14, 15).

Existing research predominantly focuses on the nephrotoxicity of individual chemotherapeutic agents or specific tumor types. Systematic analysis of the clinical characteristics and independent risk factors for CRI under multifactorial interactions remains insufficient, particularly regarding the risk of CRI in patients with comorbidities such as hypertension, diabetes, and chronic kidney disease. Therefore, this retrospective analysis of clinical data from 152 malignant tumor patients undergoing chemotherapy investigates the clinical characteristics of CRI, changes in renal function before and after chemotherapy, and associated factors. The aim is to generate hypotheses and provide preliminary evidence that may inform future prospective studies aimed at developing individualized prevention and intervention strategies.

## Materials and methods

### Study population

Patients with malignant tumors undergoing chemotherapy in the Oncology Department of our hospital from January 2020 to December 2023 were enrolled as study subjects. Inclusion Criteria: (1) Pathologically confirmed malignant tumor via histology or cytology; (2) Completion of at least one full cycle of systemic chemotherapy; (3) Complete clinical records including general information, tumor-related details, chemotherapy regimen, and renal function indicators before and after treatment; (4) No history of established renal failure prior to chemotherapy, or presence of mild renal impairment deemed clinically tolerable for chemotherapy. Exclusion Criteria: (1) Pre-chemotherapy diagnosis of end-stage renal disease requiring regular dialysis; (2) Concurrent conditions potentially causing renal injury (e.g., acute infection, autoimmune disease, urinary tract obstruction); (3) Use of non-chemotherapy nephrotoxic agents (e.g., aminoglycoside antibiotics, NSAIDs) during the chemotherapy period; Importantly, the use of nephrotoxic chemotherapeutic agents (e.g., cisplatin, ifosfamide) was not an exclusion criterion but was analyzed as a potential risk factor for CRI; (4) Incomplete clinical data (e.g., missing key laboratory parameters or medical history records) preventing statistical analysis.

Following rigorous screening based on inclusion and exclusion criteria, 152 patients with malignant tumors were ultimately enrolled. They were divided into the CRI group (chemotherapy-induced renal injury, *n* = 28) and the Non-CRI group (no renal injury, *n* = 124) based on the occurrence of CRI.

### Criteria for renal injury diagnosis

Chemotherapy-related renal injury (CRI) was defined as a composite endpoint comprising either acute kidney injury (AKI) occurring within 1 week after the first chemotherapy cycle, or chronic kidney disease (CKD) persisting for more than 3 months following chemotherapy.

AKI was defined according to the KDIGO criteria: ① Serum creatinine increase ≥ 26.5 μmol/L within 48 hours; ② Serum creatinine increase ≥ 1.5 times baseline within 7 days; ③ Urine output < 0.5 mL/(kg⋅h) for ≥ 6 h.

CKD was defined as renal dysfunction persisting for more than 3 months after chemotherapy, including: ① Persistent elevation of serum creatinine; ② eGFR < 60 mL/(min⋅1.73 m^2^); ③ Persistent proteinuria.

The timing of renal function assessment was standardized for all patients based on the first post-chemotherapy evaluation (within 1 week after cycle 1) and at the 3-month follow-up to distinguish AKI from CKD and to clarify the temporal relationship between chemotherapy exposure and renal outcomes. This standardized approach ensured consistency in outcome ascertainment across the study population.

### Data collection

The following patient information was retrospectively collected via the hospital’s electronic medical record system: patient age, gender, body mass index (BMI), smoking history, alcohol consumption history, hypertension (previously diagnosed or blood pressure ≥ 140/90 mmHg at admission), diabetes (previously diagnosed or fasting blood glucose ≥ 7.0 mmol/L), history of chronic kidney disease (previously diagnosed or estimated glomerular filtration rate eGFR < 60 mL/min/1.73 m^2^ for ≥ 3 months), tumor type (e.g., lung cancer, gastric cancer, colorectal cancer, breast cancer, etc.), tumor stage (classified as Stage I-II or Stage III-IV according to the TNM staging system); Whether nephrotoxic agents (e.g., cisplatin, ifosfamide, high-dose methotrexate) are included in the chemotherapy regimen; number and types of chemotherapy drugs administered; and serum creatinine (SCr, μmol/L), blood urea nitrogen (BUN, mmol/L), uric acid (UA, μmol/L), and estimated glomerular filtration rate (eGFR, mL/min/1.73 m^2^). Given the retrospective nature of data collection, the completeness and accuracy of the recorded information cannot be guaranteed, and potential documentation biases may exist.

### Statistical analysis

Data were analyzed using GraphPad Prism 9.50 software (GraphPad Software Inc., San Diego, CA, United States) and SPSS 27.0 statistical software (SPSS, Inc., Chicago, IL, United States). Data normality was assessed using the Shapiro-Wilk test. For normally distributed quantitative data, results are presented as mean ± standard deviation (x ± s), with comparisons between groups performed using the independent samples *t*-test. For non-normally distributed quantitative data, results are presented as median (interquartile range) [M(Q1,Q3)], with comparisons performed using the Mann-Whitney U test. Categorical data were expressed as counts and percentages (n, %). Intergroup comparisons were performed using the Chi-square test. “Whether CRI occurred” was designated as the dependent variable. Univariate logistic regression was performed for all candidate variables. Given the limited number of CRI events (*n* = 28), only variables with *P* < 0.05 in univariate analysis were considered for multivariate analysis to avoid model overfitting. A maximum of one predictor per 5–10 events was maintained to ensure estimate stability, resulting in the inclusion of six covariates in the final model. Stepwise selection was used for variable entry. Model diagnostics, including the Hosmer–Lemeshow goodness-of-fit test (*P* = 0.672) and variance inflation factors (all < 2.5), indicated adequate model fit and no significant multicollinearity. Independent risk factors were selected using stepwise regression. All *P*-values were derived from two-sided tests, with *P* < 0.05 indicating statistical significance.

## Results

### Comparison of general characteristics between patient groups

This study included 152 patients with malignant tumors undergoing chemotherapy. Based on the occurrence of chemotherapy-related renal injury (CRI) (CRI), patients were divided into the Non-CRI group (no CRI, *n* = 124) and the CRI group (CRI occurrence, *n* = 28). The comparison of general characteristics between the two groups is shown in Table 1: Comparisons of general characteristics such as gender, BMI, smoking history, and alcohol consumption history showed no statistically significant differences between groups (*P* > 0.05). Regarding age distribution, the proportion of patients aged ≥ 60 years was significantly higher in the CRI group (67.86%) than in the Non-CRI group (40.32%), with a statistically significant difference (χ^2^ = 6.986, *P* = 0.008). Regarding underlying conditions, the CRI group exhibited significantly higher prevalence of hypertension (53.57%), diabetes (46.43%), and chronic kidney disease history (17.86%) compared to the Non-CRI group (28.23%, 21.77%, 4.03%), with all differences statistically significant (all *P* < 0.05).

**TABLE 1:** Comparison of general characteristics between the two groups of patients.

Indicator	Non-CRI group(*n* = 124)	CRI group(*n* = 28)	χ^2^/t	*P*-Value
Gender (n,%)				
Male	73(58.87%)	16(57.14%)	0.028	0.867
Female	51(41.13%)	12(42.86%)		
Age (n,%)				
≤ 60 years	74(59.68%)	9(32.14%)	6.986	0.008
> 60 years	50(40.32%)	19(67.86%)		
BMI(kg/m^2^)	22.62 ± 1.35	22.79 ± 1.43	0.606	0.545
Smoking history (n,%)	40(32.26%)	10(35.71%)	0.124	0.725
Drinking history (n,%)	29(23.39%)	8(28.57%)	0.333	0.564
Hypertension (n,%)	35(28.23%)	15(53.57%)	6.648	0.010
Diabetes (n,%)	27(21.77%)	13(46.43%)	7.160	0.008
Chronic kidney disease history (n,%)	5(4.03%)	5(17.86%)	7.103	0.008

BMI, Body Mass Index. Count data are expressed as case numbers and percentages, analyzed using the Chi-square test. For normally distributed continuous data, results are presented as mean ± standard deviation, with intergroup comparisons performed using the independent samples *t*-test. *P* < 0.05 indicates statistically significant differences.

### Comparison of tumor types and chemotherapy-related data between the two groups

There were no statistically significant differences between the two groups in the distribution of tumor types (lung cancer, gastric cancer, colorectal cancer, breast cancer, etc.) or tumor stages (Stage I–II, Stage III–IV) (all *P* > 0.05). Chemotherapy-related indicators showed that the rate of nephrotoxic chemotherapy drug use in the CRI group was 82.14%, significantly higher than the 55.65% in the Non-CRI group (χ^2^ = 6.713, *P* = 0.010). Regarding the number of chemotherapy drugs, the proportion of patients receiving ≥ 3 drugs was 57.14% in the CRI group, significantly higher than the 28.23% in the Non-CRI group (χ^2^ = 8.833, *P* = 0.012) (Table 2).

**TABLE 2:** Comparison of tumor types and chemotherapy-related data between the two patient groups.

Indicator	Non-CRI group (*n* = 124)	CRI group (*n* = 28)	χ^2^	*P*-value
Tumor type (n,%)				
Lung cancer	40(32.26%)	12(42.86%)	1.431	0.839
Gastric cancer	35(28.23%)	6(21.43%)		
Colorectal cancer	27(21.77%)	5(17.86%)		
Breast cancer	15(12.10%)	3(10.71%)		
Other tumors	7(5.65%)	2(7.14%)		
Tumor stage (n,%)				
Stage I–II	33(26.61%)	6(21.43%)	0.322	0.571
Stage III–IV	91(73.39%)	22(78.57%)		
Use of nephrotoxic chemotherapy agents	69(55.65%)	23(82.14%)	6.713	0.010
Number of chemotherapy agents (n,%)				
1 agent	38(30.65%)	4(14.29%)	8.833	0.012
2 agents	51(41.13%)	8(28.57%)		
≥ 3 agents	35(28.23%)	16(57.14%)		

Count data are expressed as case numbers and percentages, analyzed using the Chi-square test. *P* < 0.05 indicates statistically significant differences.

### Changes in renal function indicators before and after chemotherapy in both groups

**FIGURE 1:**
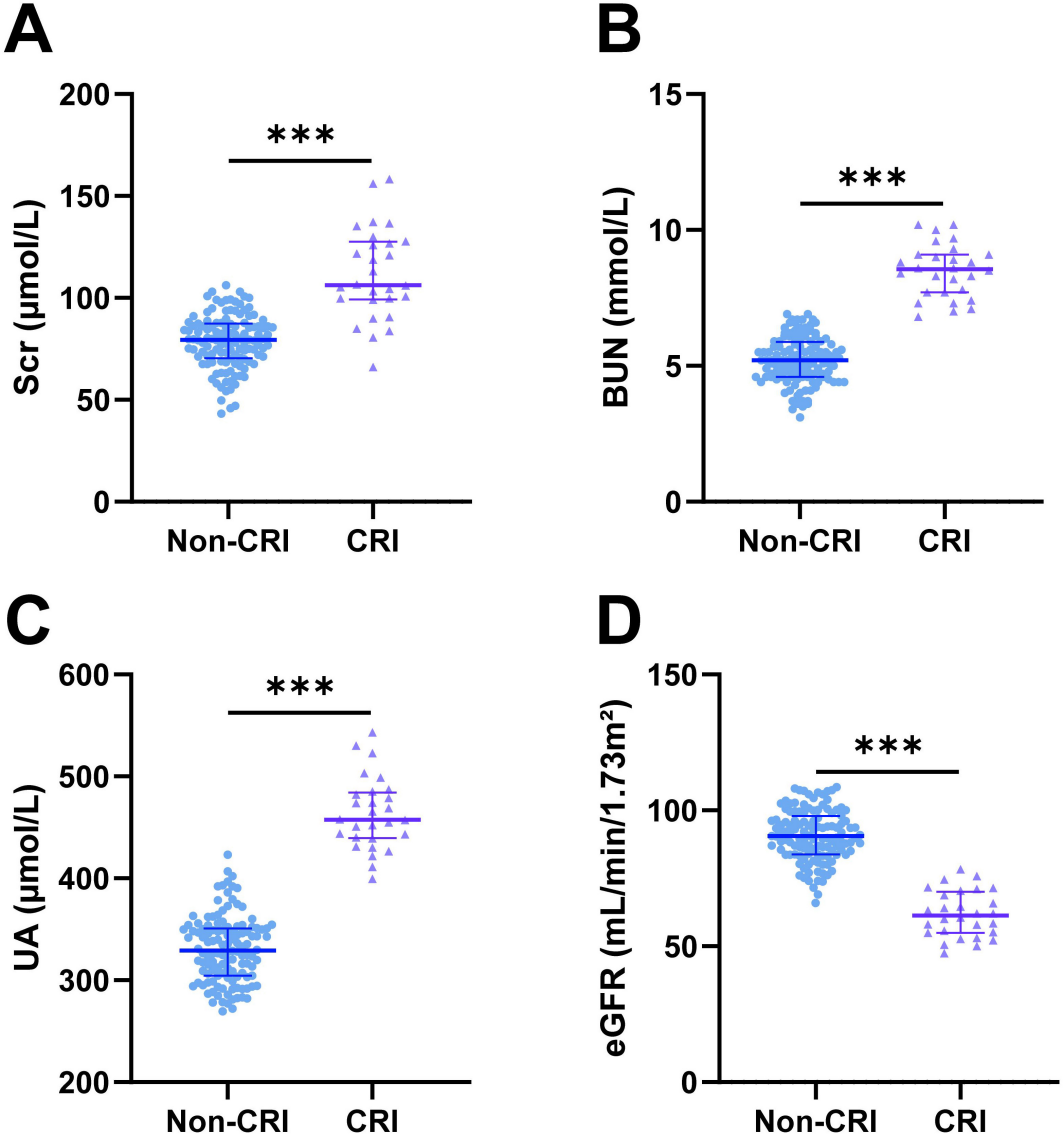
Comparison of changes in renal function indicators between two groups of patients after chemotherapy. **(A)** SCr: Serum Creatinine. **(B)** BUN: Blood Urea Nitrogen. **(C)** UA: Uric Acid. **(D)** eGFR: Estimated Glomerular Filtration Rate. *P* < 0.05 indicates statistically significant differences. *** denotes *P* < 0.001.

Before chemotherapy, comparisons of serum creatinine (SCr), blood urea nitrogen (BUN), uric acid (UA), and estimated glomerular filtration rate (eGFR) levels between the two groups showed no statistically significant differences (all *P* > 0.05) (Table 3). After chemotherapy, renal function parameters in the Non-CRI group remained relatively stable. In contrast, patients in the CRI group exhibited marked renal impairment, with significant increases in SCr, BUN, and UA, and a decrease in eGFR compared to the Non-CRI group (all *P* < 0.001) (Figure 1 and Table 3). The mean percentage changes in the CRI group were as follows: SCr increased by 45.1%, BUN by 64.5%, UA by 40.8%, and eGFR decreased by 33.1%, indicating substantial renal function deterioration.

**TABLE 3:** Comparison of changes in renal function indicators before chemotherapy between the two groups of patients.

Renal function indicators	Non-CRI group	CRI group	*t*	*P*
SCr (μmol/L)	74.73 ± 11.93	76.98 ± 12.18	0.894	0.373
BUN (mmol/L)	4.91 ± 0.71	5.16 ± 0.78	1.635	0.104
UA (μmol/L)	325.50 ± 28.31	329.10 ± 30.52	0.598	0.551
eGFR (ml/min⋅1.73 m^2^)	94.22 ± 8.53	92.40 ± 8.33	1.028	0.306

SCr, Serum Creatinine; BUN, Blood Urea Nitrogen; UA, Uric Acid; eGFR, Estimated Glomerular Filtration Rate. For normally distributed quantitative data, results are expressed as mean ± standard deviation. Comparisons between groups were performed using the independent samples *t*-test. *P* < 0.05 indicates statistically significant differences.

### Univariate logistic regression analysis for CRI occurrence

Using “whether CRI occurred” as the dependent variable (did not occur = 0, occurred = 1), univariate logistic regression analysis was performed with the following independent variables: gender, age, BMI, smoking history, alcohol consumption history, underlying comorbidities (history of hypertension, diabetes, chronic kidney disease), tumor type, tumor stage, use of nephrotoxic chemotherapy agents, and number of chemotherapy drugs as independent variables (Table 4) to conduct univariate logistic regression analysis, establishing preliminary associations between these factors and CRI occurrence. Results (Table 5) showed that age ≥ 60 years, comorbid hypertension, diabetes, chronic kidney disease history, use of nephrotoxic chemotherapy agents, and increased number of chemotherapy agents were significantly associated with elevated risk of CRI (all *P* < 0.05). Gender, BMI, smoking history, alcohol consumption history, tumor type, and tumor stage were not significantly associated with CRI occurrence (all *P* > 0.05).

**TABLE 4:** Assignment table for research variables.

Indicators	Variable type	Assignment specifications
Gender	Binary variable	Male = 1, Female = 0
Age	Binary variable	> 60 years old = 1, ≤ 60 years old = 0
BMI	Continuous variable	Measured Value (kg/m^2^)
Smoking history	Binary variable	Present = 1, Absent = 0
Alcohol consumption history	Binary variable	Present = 1, Absent = 0
Hypertension	Binary variable	Present = 1, Absent = 0
Diabetes	Binary variable	Present = 1, Absent = 0
History of chronic kidney disease	Binary variable	Present = 1, Absent = 0
Tumor type	Binary variable	Lung Cancer = 1, Other Types = 0
Tumor stage	Binary variable	Stage III–IV = 1, Stage I–II = 0
Use of nephrotoxic chemotherapy drugs	Binary variable	Present = 1, Absent = 0
Number of chemotherapy drugs	Continuous variable	Number of Actual Medications Used

**TABLE 5:** Univariate logistic regression analysis for cri occurrence.

Indicators	Regression coefficient (β)	Standard error (SE)	OR-value	95%CI	*P-*value
Gender	−0.071	0.423	0.932	0.406∼2.135	0.867
Age	1.139	0.444	3.124	1.308∼7.461	0.010
BMI	0.094	0.154	1.098	0.812∼1.485	0.543
Smoking history	0.154	0.439	1.167	0.494∼2.757	0.725
Alcohol consumption history	0.270	0.469	1.310	0.523∼3.286	0.564
Hypertension	1.076	0.428	2.934	1.267∼6.792	0.012
Diabetes	1.136	0.437	3.114	1.322∼7.332	0.009
History of chronic kidney disease	1.644	0.672	5.174	1.386∼19.320	0.014
Tumor type	0.454	0.427	1.575	0.681∼3.640	0.288
Tumor stage	0.285	0.503	1.330	0.496∼3.566	0.571
Use of nephrotoxic chemotherapy drugs	1.299	0.526	3.667	1.309∼10.270	0.013
Number of chemotherapy drugs	0.789	0.254	2.202	1.340∼3.620	0.002

### Multivariate logistic regression analysis for CRI occurrence

Variables with *P* < 0.05 in univariate logistic regression analysis [age, comorbidities (hypertension, diabetes, history of chronic kidney disease), use of nephrotoxic chemotherapy agents, and number of chemotherapy agents] were incorporated into a multivariate logistic regression model to identify independent risk factors for CRI occurrence. Results showed: Age ≥ 60 years (OR = 3.277, 95% CI: 1.175–9.134, *P* = 0.023), concurrent diabetes (OR = 4.544, 95% CI: 1.612–12.809, *P* = 0.004), history of chronic kidney disease (OR = 6.348, 95% CI: 1.100–36.638, *P* = 0.039), use of nephrotoxic chemotherapy agents (OR = 3.930, 95% CI: 1.143–13.511, *P* = 0.030), and increased number of chemotherapy agents (OR = 2.068, 95% CI: 1.185–3.612, *P* = 0.011) were independent risk factors for CRI occurrence (all *P* < 0.05) (Table 6).

**TABLE 6:** Multifactorial logistic regression analysis of CRI occurrence.

Indicators	Regression coefficient (β)	Standard error (SE)	OR-value	95%CI	*P*-value
Age	1.187	0.523	3.277	1.175∼9.134	0.023
Hypertension	0.585	0.530	1.795	0.635∼5.075	0.270
Diabetes	1.514	0.529	4.544	1.612∼12.809	0.004
History of chronic kidney disease	1.848	0.894	6.348	1.100∼36.638	0.039
Use of nephrotoxic chemotherapy drugs	1.369	0.630	3.930	1.143∼13.511	0.030
Number of chemotherapy drugs	0.727	0.284	2.068	1.185∼3.612	0.011

## Discussion

Currently, the incidence of malignant tumors remains persistently high. While chemotherapy, as a core treatment modality, effectively prolongs patient survival, its toxic effects on normal organs cannot be overlooked (16, 17). As a key organ for drug metabolism and excretion, the kidneys are highly susceptible to the impact of chemotherapeutic agents, leading to chemotherapy-related renal injury (CRI) (18). Particularly with the increasing prevalence of multi-agent combination chemotherapy and the rising proportion of elderly cancer patients, the risk of CRI has significantly increased. This complication not only may necessitate interruption of chemotherapy regimens or dose adjustments, thereby compromising antitumor efficacy, but may also further induce AKI, CKD, or even end-stage renal disease, substantially exacerbating patients’ health impairment and medical burden (19, 20). Given this, this study retrospectively analyzed clinical data from 152 malignant tumor patients undergoing chemotherapy to systematically investigate the occurrence patterns, clinical characteristics, and factors associated with CRI. The aim is to provide preliminary evidence that may inform early identification of high-risk populations, optimization of renal protection strategies, and development of individualized intervention measures in future prospective studies.

Among the independent risk factors for CRI, age ≥ 60 years (OR = 3.277) represents a significant physiological factor. This finding aligns with multiple prior studies, potentially due to reduced nephron number, naturally declining estimated glomerular filtration rate (eGFR), and diminished renal blood flow regulation in elderly patients. Combined with frequent comorbidities and polypharmacy, these factors significantly impair renal compensation capacity against chemotherapy drug toxicity (21–23). Therefore, when formulating chemotherapy regimens for elderly cancer patients, their renal reserve capacity should be thoroughly assessed. Priority should be given to selecting drugs with lower nephrotoxicity (e.g., carboplatin instead of cisplatin) or adjusting dosages based on renal function.

Regarding underlying conditions, this study confirmed that diabetes mellitus (OR = 4.544) and a history of chronic kidney disease (OR = 6.348) are high-risk factors for CRI. Although hypertension was associated with CRI in univariate analysis, it did not enter the multivariate regression model. It is speculated that hypertension may indirectly influence CRI occurrence through synergistic effects with other factors, such as diabetes. Diabetes itself can induce diabetic nephropathy through mechanisms including accumulation of advanced glycation end products, enhanced oxidative stress, and glomerular hyperfiltration, placing the kidneys in a state of “subclinical injury” (24). Against this backdrop, chemotherapy drugs (such as cisplatin and methotrexate) further exacerbate damage to tubular epithelial cells, readily triggering acute or chronic renal deterioration (25, 26). In this study, the prevalence of diabetes in the CRI group reached 46.43%, significantly higher than the Non-CRI group (21.77%). This underscores the need for enhanced dynamic renal function monitoring before and after chemotherapy in diabetic patients, with protective measures such as hydration and urine alkalization implemented when necessary. Patients with chronic kidney disease already possess insufficient renal reserve and persistently low eGFR levels. The accumulation of chemotherapy drugs and their metabolites within the body further exacerbates parenchymal damage (27). This aligns with the finding that the proportion of CRI patients with a history of chronic kidney disease (17.86%) was significantly higher than that in the Non-CRI group (4.03%). This observation suggests that stricter renal protection measures are required during chemotherapy for patients with such underlying conditions.

Among chemotherapy regimen-related factors, the use of nephrotoxic chemotherapy drugs (OR = 3.930) and an increased number of chemotherapy drugs (OR = 2.068) were key risk factors for CRI occurrence. The CRI group exhibited significantly higher rates of nephrotoxic chemotherapy drug use (82.14%) and the proportion of patients receiving ≥ 3 chemotherapy drugs (57.14%) compared to the Non-CRI group (55.65 and 28.23%, respectively). This finding confirms the nephrotoxicity inherent to chemotherapy drugs themselves and the additive effects of multi-drug combinations. Classic nephrotoxic agents such as cisplatin, ifosfamide, and high-dose methotrexate remain widely used in solid tumor treatment. They cause renal injury through mechanisms including direct damage to renal tubular epithelial cells, induction of inflammatory responses, and oxidative stress (28–30). While multi-drug combinations enhance antitumor efficacy, they also increase drug interactions and renal metabolic burden, providing crucial guidance for clinical chemotherapy regimen design (4). This study demonstrates that patients receiving ≥ 3 chemotherapeutic agents exhibit a significantly elevated risk of CRI, suggesting that regimens should be simplified and unnecessary multi-drug combinations avoided whenever possible while maintaining therapeutic intensity—particularly in elderly patients or those with comorbidities.

Comparative analysis of renal function metrics before and after chemotherapy between the two groups further substantiated CRI’s nephrotoxic effects: the CRI group exhibited significant post-chemotherapy elevations in serum creatinine, blood urea nitrogen, and uric acid, alongside marked decreases in eGFR, whereas the Non-CRI group showed no notable abnormal fluctuations. These findings suggest that regular monitoring of renal function indicators (particularly serum creatinine and eGFR) during chemotherapy can facilitate timely detection of renal injury, providing a basis for early intervention.

This study has several limitations that should be considered when interpreting the findings. First, its retrospective design and single-center setting may introduce selection bias and limit the generalizability of the findings. The lack of standardized data collection inherent to retrospective studies also increases the risk of information bias. Furthermore, although renal function assessments were performed at standardized time points (within 1 week post-cycle 1 and at 3 months), the exact timing of serum creatinine measurements relative to chemotherapy administration may have varied slightly due to the retrospective nature of data collection, potentially influencing the detection of AKI events. Second, the relatively small number of CRI events (*n* = 28) significantly restricted the statistical power of the analysis. This small sample size limited the number of variables that could be included in the multivariate model, increasing the risk of overfitting and contributing to the wide confidence intervals observed for some predictors (e.g., history of chronic kidney disease: 95% CI 1.100–36.638), which suggests instability in the effect estimates. Although the Hosmer-Lemeshow test indicated acceptable model fit (*P* = 0.672), these findings should be interpreted with caution, and validation in larger cohorts is warranted. Third, detailed data on potential confounders-such as cumulative chemotherapy doses, hydration protocols, use of renal protective agents (e.g., amifostine), exposure to contrast media, and concurrent medications affecting renal function-were not available, which may introduce residual confounding. Additionally, the heterogeneity of tumor types and chemotherapy regimens precluded detailed subgroup analyses, and variations in supportive care practices may have influenced renal outcomes. Fourth, the heterogeneity of tumor types and chemotherapy regimens limits the feasibility of detailed subgroup analysis. Moreover, the lack of external validation from multicenter data means that these findings may not be applicable to other institutions with different chemotherapy protocols or supportive care practices. Fifth, long-term outcomes, including renal function recovery or progression to end-stage renal disease, were not assessed. Additionally, although renal function assessments were conducted at standardized time points (within 1 week post-cycle 1 and at 3 months), the lack of more frequent monitoring between these time points may have resulted in under-detection of transient AKI episodes that resolved prior to the scheduled assessments. Given these methodological constraints, the identified risk factors should be viewed as exploratory rather than definitive. Prospective studies with larger, multicenter cohorts and more granular data collection are warranted to validate these findings and establish causality.

In summary, this study identifies several factors associated with CRI after chemotherapy in cancer patients, including age, comorbidities, and chemotherapy regimens. However, due to the observational nature of the study, these findings should be interpreted as associations rather than causal relationships. The use of standardized time points for renal function assessment strengthens the validity of our outcome classification, although the inherent limitations of retrospective data collection remain. However, owing to the retrospective design and limited sample size, these findings should be interpreted as preliminary and hypothesis-generating rather than definitive. If validated in future prospective studies, these associations may inform clinical strategies such as enhanced baseline renal assessment for patients aged ≥ 60 years or those with diabetes or chronic kidney disease, prioritization of agents with lower nephrotoxicity, and cautious use of multi-drug regimens. Until such validation is available, clinicians should continue to exercise clinical judgment based on established guidelines. Dynamic monitoring of renal function indicators during chemotherapy remains a prudent approach to enable early identification of abnormalities and timely intervention, with the goal of balancing tumor treatment and renal protection.

## Data Availability

The original contributions presented in the study are included in the article/Supplementary material, further inquiries can be directed to the corresponding authors.
